# Positron emission tomography: An overview

**DOI:** 10.4103/0971-6203.25665

**Published:** 2006

**Authors:** A. K. Shukla, Utham Kumar

**Affiliations:** Department of Nuclear Medicine, Sanjay Gandhi Post Graduate Institute of Medical Sciences, Lucknow, Uttar Pradesh, India

**Keywords:** Bismuth germinate (BGO), Flurodeoxyglucose (FDG), Lutetium oxyorthosilicate (LSO), Positron emission tomography

## Abstract

The rate of glucose utilization in tumor cells is significantly enhanced as compared to normal cells and this biochemical characteristic is utilized in PET imaging using FDG as a major workhorse. The PET systems as well as cyclotrons producing positron emitting radiopharmaceuticals have undergone continuous technological refinements. While PET (CT) systems enable fusion images as well as precise attenuation correction, the self-shielded cyclotrons developed provide dedicated systems for in-house production of a large number of PET radiopharmaceuticals. The application of PET images in oncology includes those of pulmonary, colorectal, breast, lymphoma, head & neck, bone, ovarian and GI cancers. The PET has been recognized as promising diagnostic tool to predict biological and physiological changes at the molecular level and hence offer a potential area for future applications including Stem Cell research.

## Introduction

Much of the enthusiasm concerning fast emerging applications of Positron Emission Tomography can be attributable to its clear-cut recognition as a unique imaging modality with proven clinical value in the management of cancer patients. Unlike other imaging modalities, Positron Emission Tomography (PET) quantitatively demonstrates scintigraphic representation of tumor physiology as well as anatomy and thus assumes unique advantage over other cross-sectional imaging methods such as Computed Tomography (CT). In order to permit tumor imaging using PET or any other tracer technique, the basic features deployed relate to differences in physiological and metabolic characteristics of tumor and normal tissues. These differences include tumor cell surface antigen phenotype versus normal tissues. In general, growth and hence use of DNA precursors such as thymidine as well as rates of protein synthesis in tumors are often increased as compared to normal tissues. Transport and incorporation of various types of amino acids as well as rates of anaerobic and aerobic glycolysis has been observed in tumor cells.[1] In a wide range of tumor types, the rates of glucose utilization are significantly enhanced as compared to normal tissues. Such metabolic alterations are more pronounced in relatively aggressive tumors, even though not specific for cancer as such. However, they exhibit sufficient specificity to make it a specific feature depicting considerable clinical utility. The altered metabolism of tumor cells can be detected by using radiopharmaceuticals labeled with positron emitting radionuclide. One of the most striking biochemical characteristics that are utilized in tumor imaging using PET is the preferential consumption of glucose by the tumor cells. An analog of 2-deoxyglucose, i.e, FDG with F-18 as a positron emitting label is the major workhorse used in tumor imaging. The increased accumulation of FDG in cancer cells has been shown to be due to (i) enhanced expression of glucose transporter molecules on the tumor cells and (ii) increased concentration and/or action of hexokinase.

## Basic principles

**Figure 1:**
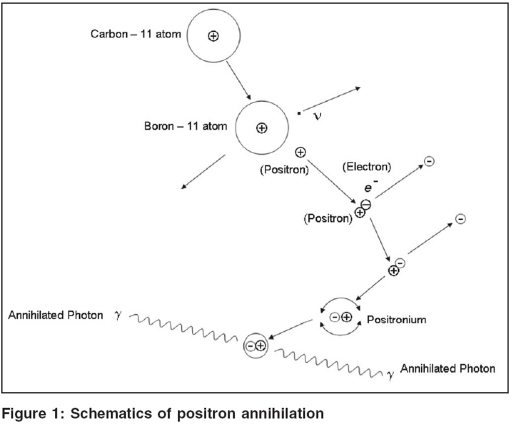
Schematics of positron annihilation

A positron is an antiparticle of an electron with identical mass and charge. After emission, the positron has some kinetic energy, which is lost through multiple collisions with electrons present in the neighboring tissues. The complete or almost complete loss of energy by the positron results into its combination with electron. This eventually forms a short-lived composition, i.e., positronium. The schematics are shown in Figure 1. The positronium thus created, being short-lived, eventually gets annihilated, converting all its mass into energy and thereby emitting two photons of 511 keV each (which is resting energy of the electron or positron) in opposite direction as depicted in the Figure 1. This ensures conservation of energy and momentum. The unique characteristic of simultaneous emission of two annihilated photons forms the basis for detection and localization of positron emitters using a novel technique called coincidence detection.[2] Scintillation detectors - e.g., bismuth germinate (BGO) or Lutetium Oxyorthosilicate (LSO) - and photomultiplier tubes are placed opposite to the source of positron emitter. The signals are then fed into separate amplifiers and energy discriminating circuits. This process results into detection of a coincidence event, which localizes an annihilation event somewhere along the line joining the two detectors. In a typical PET scanner, there are hundreds of such points of detector banks in the form of ring surrounding the patient. It can, therefore, be stated that the PET scanning in a comprehensive manner relates to detection of millions of coincidence events and hence provides information about the concentration and spatial location of positron emitters within the patient.

## PET image formation

Each pair of parallel and opposite detectors produces a coincidence line, which is unique in terms of location and direction. A large number of such coincidence lines form the data set and by the use of which a cross-sectional image can be reconstructed. The data pertaining to coincidence events is stored as two-dimensional matrix in which horizontal direction represents offset from the center of the field of view (CFOV), whereas vertical direction describes the projection angle. This set of data in terms of two-dimensional matrix is called ‘Sinogram’ and provides a set of projection data for reconstruction of image. Sinogram data however needs to be corrected for tissue alternations as well as detector non-uniformities. Various detector elements in a PET system are expected to exhibit variation in detection efficiency due to geometrical variation, differences in energy discrimination as well as detector gains. Such variations need to be equalized to prevent appearance of any artifacts. In addition, attenuation correction accounts for the compensation due to intra-tissue absorption of one or both annihilated photons. After necessary corrections, the Sinogram cumulatively represents all the coincidence events along a particular coincidence line. Sinogram data is then used to reconstruct the image using filtered back projection or an interactive technique.

## Types of coincidence events

**Figure 2:**
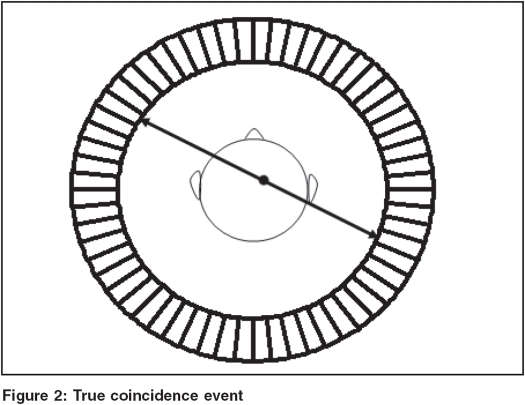
True coincidence event

**Figure 3:**
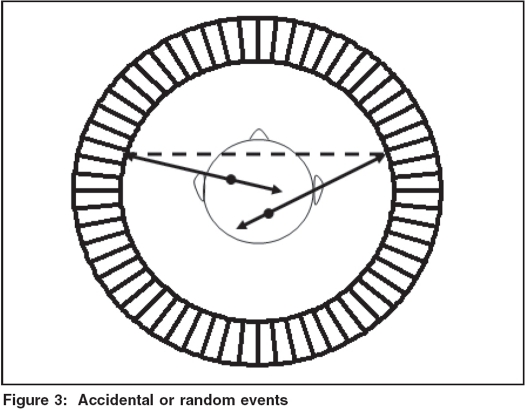
Accidental or random events

Ideally speaking, PET scanner should record only those coincidence events that originate as a result of positron annihilation along the line between the two parallel opposite detectors. Such detected events are called True Coincidences as depicted in Figure 2 and they carry information as regards the spatial location of the positron source. In case of true coincidences, the detector pair should ideally produce signals simultaneously but due to limitations of scintillation detector as well as associated electronics, coincidence events are accepted within a finite interval, which is of the order of 4 to12 ns. It is for this reason that two unrelated photons may be detected and get registered as coincidence events. Such a situation is shown in Figure 3 and related events so detected are called Accidental or Random Events and in reality do not carry any useful information regarding spatial location of activity distribution and are undesirable and need to be corrected for.[3] The noise due to accidental events may be more pronounced at higher count rates and eventually cause increased background in the final images.

**Figure 4:**
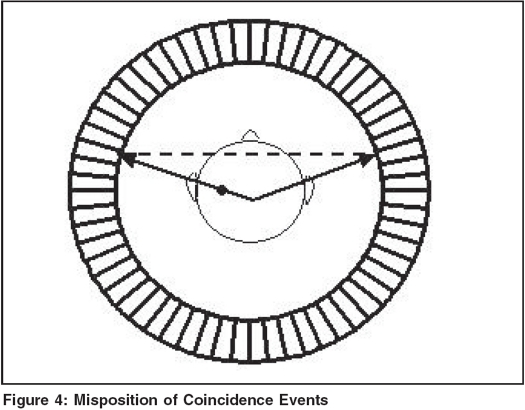
Misposition of Coincidence Events

In addition to accidental or random events, there may be a situation where both of the annihilated photons are diverted from their original direction as a result of Compton interaction and reach the detector to misposition the coincidence event as shown in Figure 4. Such events are called Scatter Coincidences. In a typical situation, scatter coincidences contribute approximately 40% of the total recorded coincidences and this may vary depending on the size of the object. The scatter events are likely to affect overall contrast of the PET image. However, such scatter events can be discriminated from true events on the basis of energy of the scattered photons. The scatter events can therefore be effectively eliminated by using lead or tungsten placed between the imaging slices.[4] For this purpose, an interplane septa of 1 mm. thickness and approximately 15 cm length is good enough to eliminate scatter events originating from the regions outside a particular slice.

In an ideal situation, only two detectors in a coincidence time window would be required to be activated. However, at higher count rates, more than one positron gets annihilated and more than two detectors may get activated. This results into ambiguity about the location of the event. Such ambiguous events can be distinguished from valid coincidence events and are usually discarded and hence result into net reduction in overall count rate.

## Intra-patient absorption and attenuation correction

As already described, in a positron annihilation two photons are simultaneously emitted in opposite directions. If either photon is absorbed within the body, a coincidence event will not take place. Such a probability of attenuation of photon within the body exists also for all other annihilation events independent of the position. A blank scan followed by transmission scan for all possible coincidence lines using a rod or point source (that rotates around the patient) enables measurement of transmission count rates. However, transmission scan can also be performed after tracer administration.[5,6] The transmission source used is usually 68 Ge, a positron emitter having a half life of 275 days. The degree of absorption in PET is incidentally more as compared to SPECT (140 keV) even though energy in the former case is much higher, i.e, 511 keV. This is simply because the annihilated photons traverse entire body rather than just the distance from the detector. The present day PET systems are now combining with multislice CT system to enable not only precise attenuation correction but also provide a tool to generate fusion images through co-registration.

## PET detectors

In view of the fact that emitted photons to be detected in PET have energy of 511 KeV, the detectors to be used need to have specialized characteristics. These include stopping power, amount of light produced against each absorbed photon and time taken for the decay of light.

The increased stopping power enables detector's ability to efficiently absorb the total energy of 511 keV of annihilated photons. In order to achieve this, a scintillator with high effective atomic number and linear attenuation coefficient needs be used. This would enable predominantly the photoelectric interactions within the crystal or scintillator and impart full absorption of energy.

In addition, the detector should also have a capability of producing higher light output so as to have better energy resolution. The increased light output also enhances accuracy of detector element configuration. Also the decay time of the light decides how accurately coincident photons can be detected by a pair of detectors. The shorter time constant enables faster production of the signal after complete absorption. With fast scintillators, use of narrow coincidence time window would suffice and would reduce the probability of detection of unrelated or accidental coincident events. The signal is integrated for three or four times the decay constant and during this period, the detector is dead. This dead time of the system is a main factor limiting count rates, particularly at high activity levels in PET systems.

## Choice of pet scintillator

Bismuth Germanate (BGO) has been predominantly used as a detector of choice for the last several years because it possesses the desired characteristics, namely, high effective atomic number as well as high density and hence high stopping power. Approximately, 95% of the annihilated photons of 511 keV undergo interaction within a 3-cm thick block of BGO detector. On the contrary, only 36% of the emitted photons undergo interaction in the 3-cm block of NaI detector. However, the light output of BGO detector is fairly poor as compared to NaI detector. In addition, BGO has longer decay time constant and therefore enhanced dead time and hence limits the count rate performance.

Recently, Lutetium Oxyorthosilicate (LSO) has been identified to possess better characteristics and hence is ideally suited for use as a scintillating material (detector) in PET.[7] It provides considerably higher light output and hence permits development of high resolution block detector systems with bare minimum loss of spatial resolution. The decay time constant is also shorter so as to have reduced dead time and allow use of shorter coincidence time window, thereby drastically curtailing the random events at higher activity levels.

Table 1 depicts characteristics of three scintillators, namely, Sodium Iodide (NaI), Bismuth Germanate (BGO) and Lutetium Oxyorthosilicate (LSO).

**Table 1:** Comparative characteristics of PET scintillators

*Characteristic/parameter*	*Bismuth germanate (BGO)*	*Lutetium oxyorthosilicate (LSO)*	*Sodium iodide NaI (Tl)*
Crystal Density(gm/cc)	7.13	7.40	3.67
Eff. Atomic Number (Z eff.)	74	66	51
Decay Time (nano sec.)	300	40	230
Light output	15	75	100
Refractive Index	2.15	1.82	1.85

In a typical PET system, there are multiple ranges of block detectors around the patient so as to record coincidence events emanating from all possible projections around the patient. BGO/LSO detectors are stated to be the best suited for a wide range of clinical studies and improved count rate performance. However, use of LSO would further improve dynamic capability of detection in PET systems.

## Use of SPECT as PET

Even though most of the dual-head gamma cameras deploy NaI as crystal, an idea has been mooted to use the dual-head SPECT system as PET scanner, which is now a reality at many centres. Such systems can be either used as dual-head SPECT systems or as PET systems by using coincidence circuits. This is facilitated due to advanced digital electronics for prompt processing of signals from photo-multiplier tubes. In case of a dual head gamma camera being operated as PET system, pulse extrapolation local centroid positioning is used in coincidence mode. In addition, crystal thickness is required to be enhanced (about 1.5 cm) compared to 0.9 cm commonly used in conventional SPECT systems. Further inventions have revealed that use of two separate layers of scintillators, - for example, LSO (Lutetium Oxyorthosilicate) and YSO (Yttriium orthosilicate)[8] - provides better option with a moderate compromise on SPECT/PET performance characteristics. This enables emergence of hybrid systems that would have characteristics of both SPECT and PET performance. Such systems can also help reducing huge cost of the PET system, which has been considered to be a major deterrent for the centres wishing to establish a PET facility. However, use of dual-head SPECT coincidence systems are associated with the following limitations:

- Gantry design of the camera requires adequate support for extremely heavy collimators needed for 511 keV photons.
- NaI (Tl) detectors used in SPECT systems are not suited for 511 keV photons and about 1.5% of true coincidence events are only detected for 0.95 cm thick crystal used.
- Spatial resolution is also substantially compromised.

It would therefore not be always advisable to encourage the use of dual-head SPECT systems for coincidence imaging.

## Three-dimensional PET

In a typical PET system, the imaging planes are separated by a Lead or Tungsten Septa to disallow the detection of photons in one imaging plane being in coincidence with photons detected in other imaging planes. Such events need to be prevented and processing of such events would need detected imaging reconstruction algorithm that would enable reconstruction of data required from all angles in a three-dimensional domain. The deployment of 3D imaging reconstruction algorithm has revealed emergence of new generation PET system that would be capable of acquiring reconstruction data in 3D and overall counting efficiency would be drastically enhanced.[9,10] The 3D acquisition mode substantially improves the image quality even if administered activity is relatively low. It may further reduce the overall scanning time in clinical studies. However, in 3D PET, the scatter coincidence events may significantly increase and can account for approximately 40% of the total detected coincidence events as compared to 15% in 2D. The increase in scatter is because of the removal of the interplane septa in 3D. The correction in 3D PET is a complicated procedure and requires quantitative values.[11] Nevertheless, 3D PET offers tremendous potential for improving image quality as well thorough clinical studies.

## Effect of time of flight

The annihilated photons may reach the detectors at different times unless the annihilation events occur at a position that is exactly located in the middle of two coincident detectors. However, this difference in arrival time is extremely small because the photons travel with a velocity that is somewhere near the velocity of light. The time difference, therefore, in a typical situation may be less than 1 nanosecond. (ns); and therefore, this is usually ignored in conventional PET systems. However, quality of image reconstruction can be further improved if necessary correction is applied for. Furthermore, the time of flight can provide the useful clue that can be used to constrain back projection - for example, a circle of few cm radius. This might help reducing noise amplification, thereby providing improved signal-to-noise ratio in the reconstructed image.[12] The most widely used detector that can incorporate for time of flight correction appears to be LSO and may be used to develop a dedicated time of flight PET.

## Factors affecting spatial resolution in PET

The major factors that can affect the spatial resolution of PET images include size of the detector used; colinearity, or most precisely, noncolinearity of annihilating photons; and range of emitted positrons. (The Positron range is defined as a finite distance the positron is required to travel within the tissue medium before undergoing annihilation.)

### Size of detectors

A typical PET scanner incorporates hundreds of discrete detectors in the form of rings around a patient. Each pair of detectors, depending on their size, constitutes a channel. This channel width is usually 0.4-0.6 cm. Any pair of annihilated photons falling within this channel width would be detected by that particular pair of detectors and hence would be delineating specific spatial location. Any reduction in size of detectors decreases the variation in a spatial positioning within the channel width. This clearly establishes that accuracy of spatial resolution is governed by the size of individual detectors.

### Colinearity

It is a common understanding that annihilated photons travel exactly in opposite directions, but this is not always true. If at the time of annihilation, both positrons and photons do not come to a complete rest, they would have some residual momentum before undergoing annihilation. In order to conserve the momentum, the annihilated photons might have some directional component rather than being emitted exactly in opposite directions, i.e,180°. This component of noncolinearity is expected to introduce some degree of error in locating the positional location of annihilated event, thereby affecting overall image resolution. In a typical case of whole body PET scanner having 100 cm diameter, noncolinearity may deteriorate image resolution by 0.2 cm.

### Range of positrons

The positrons are emitted from the unstable nucleus with a continuous range of energies and traverse a finite distance through the medium, losing energy before undergoing annihilation. The positrons emitted from[^18^] F may have energies peaking to 0.63 MeV and therefore may have a range of 2-3 mm within the tissue.[13] The direction in which a positron travels after emission decides the degree of error to be introduced in spatial localization. In a typical case, the misposition error due to range of positrons is about 0.2 mm for the positrons emitted from ^18^F, whereas this is increased to 1.2 mm for the positrons emitted from ^15^O. The ranges of positrons and their energies from major positron emitting radionuclides are depicted in [Table 2].[14,15]

**Table 2:** Range and energy of positrons emitted by major positron emitting radionuclides

*Radionuclide*	*Maximum energy (MeV)*	*Maximum range in the tissue (mm)*
^18^F	0.63	2.6
^15^O	1.74	8.4
^3^N	1.20	5.4
^111^C	0.96	4.2

## Quantification and quality control in PET

The unique feature of coincidence detection in PET enables quantitative measurement of physiological parameters like blood flow, metabolic rate and receptor density. The count rate after attenuation correction can provide co-relative projection of absolute tracer concentration. In addition, simultaneous projection acquired in a panoramic manner provides rapid sequential images that can be used to predict dynamic variability of tracer concentration. The use of ^14^C, ^15^O, ^13^N and ^18^F stimulates the atoms used in building blocks of biological molecules and are therefore easily incorporated. These features collectively provide a unique ability to measure absolute tracer concentration and this characteristic might be used for developing *in-vivo* tracer kinetic models. The spectrum of quality control parameters for effective performance monitoring include:

- Calibration check
- Uniformity
- Spatial resolution
- Scatter fraction
- Sensitivity
- Count rate losses and randoms
- Scanner cross calibration
- Drifts in coincidence timing
- Drifts in energy thresholds
- Mechanical movement of detector rings
- Removable Septa positioning
- Laser alignment
- Attenuation correction accuracy
- Data time correction accuracy
- Scatter correction accuracy
- Random coincidence correction accuracy

The scope of this article is not to describe in detail the quality control procedures related to above parameters. However, these factors are known to make considerable impact on the image resolution, count rate capability and random/scatter events, etc.

## Radiopharmaceuticals used in PET

A significant progress has been made in the technological arena to produce PET radiopharmaceuticals. The radionuclides that are commonly used in PET imaging along with their characteristics are shown in Table 3.

**Table 3:** Characteristics of various radionuclides used in PET imaging

*Radionuclide*	*Half life*	*Average positron energy*
C-11	20.4 min	0.39 MeV
N-13	10 min	0.50 MeV
O-15	2.2 min	0.72 MeV
F-18	110 min	0.25 MeV
Cu-62	9.2 min	1.3 MeV
Ga-68	68.3 min	0.83 MeV
Rb-82	1.25 min	1.5 MeV

Hundreds of chemical compounds incorporating positron emitting radionuclides have been developed and investigated for study of a host of biochemical and physiological processes. 2-(18F) Fluoro-2-deoxyglucose (FDG) is by far the most important and widely used PET radiopharmaceutical. (15-O) water, (15-O) carbon monoxide, (13-N) ammonia (11C)-1- methionine, etc, are the other regularly used PET radiopharmaceuticals. Several other radionuclides that are currently being investigated as a source of positrons include ^76^Br, ^86^Y, ^89^Zr, ^64^Cu and ^124^.

The positron emitting radionuclides are produced in the cyclotron system that are currently available commercially. The present date cyclotrons have a tailor-made design for producing four radionuclides, namely, ^15^O, ^11^C, ^18^F, ^13^N in a dedicated manner. The major companies that are producing cyclotrons include CTI Cyclotron System, Japan Steel Works, Siemens, General Electric Medical Systems. For different radionuclides, different target materials are used to achieve the desired reaction, as depicted below in Table 4. The operating characteristics of various cyclotrons manufactured by various companies have also been depicted in Table 5.

**Table 4:** Details of target materials used in production of various positron emitting radionuclides

*Positron emitter*	*Reaction*	*Target material used*	*Remarks*
^15^O	^14^N (d,n)^15^O	Nitrogen gas	Most widely used reaction
^13^N	^15^N (p,n)^15^O or	Enriched ^15^N target	Expensive target material difficult to recycle
	^16^O (N,ρ)^13^N or	Natural water	
	^12^C (d,n)^13^N	CO_2_, CH_4_, Carbon slurry, solid graphite	Most widely used reaction
			Only BN production method at low energies
^11^C	^14^N (p,ρ)^11^C or	Nitrogen gas	Most widely used reaction
	^10^B (d,n)^11^C	B_2_O_3_	Only ^11^C production method used in 1966
^18^F	^18^O (p,n)^18^F or	^18^O - enriched water	Recycling methods available
		^20^Ne (d,a)^18^F	Ne containing trace of F_2_
			^18^F formed at low specific activity

**Table 5:** Specifications and characteristics of cyclotrons manufactured by various companies

*Manufacturer*	*Model*	*Max beam current (Photon)*	*Dual irradiation*	*Number of target ports*	*Self shielding*
CTI	RDS 111	50 μA	Yes	8μA	Yes
EBCO	TR19	>150 μA	Yes	2-8	Yes
GE	PET trace	75 μA	Optimal	3-6	Yes
	Cyclone				
IBA	10/5 Cyclone	60 μA	Yes	8	No
JSW	18/5	80 μA	Yes		No
	BC2010N	70 μA	Yes	8	No
NKK-oxford	NKK-Oxford	50-100 μA	No	-	No

The present day cyclotrons, for the sake of reducing cost and simplicity, are supplied as ‘Proton only Machine’ or ‘Dual particle machine.’ The most striking feature of such cyclotron is self-shielding, which allows access of technical staff to the cyclotron room even when the machine is operational. The shielding requirements of the walls are also achievable and the machine is no longer required to be housed in the basement. The configurations of cyclotron machines are therefore customized so as to meet the chemical requirements.

## Molecular imaging

The fusion and interdisciplinary areas like cell/molecular biology, pharmacology, medical physics, image capture techniques, nuclear medicine, chemistry, biomathematics and bioinformatics together create a novel imaging paradigm called Molecular Imaging. The overall objective in molecular imaging is aimed at addressing the issues like visualization, characterization and quantification of biological processes taking place at cellular and subcellular level within the living organisms. A molecular image is thus a representative of cellular and molecular pathways including *in vivo* mechanisms of various diseases. As opposed to classical diagnostic imaging, molecular imaging, in a unique manner, unfolds the molecular abnormalities responsible to form basis of many diseases.

The frontline issues, that are being addressed in molecular imaging/molecular medicine include:

- Evolving noninvasive *in vivo* imaging techniques depicting gene expression and protein-protein interactions
- Simultaneous monitoring of molecular events
- Cell targeting and cell trafficking
- Optimization of gene and drug therapy
- Monitoring of disease progression/therapy response at molecular pathological level

The founding principles of nuclear medicine trace forward the principles or objectives of molecular imaging. Apart from nuclear medicine, where radioactive isotopes are used to produce imaging signals. Other strategies/modalities like MRI are being adopted for molecular imaging by using molecular probes to provide image in signals other than radioactivity.

### Common probes

The most widely used and simple probes are the ones routinely used in conventional medical imaging. These include nonspecific imaging probes like contrast agents, nuclear medicine tracer (radioisotopes) and fluorochrome reporters. Such probes are effectively utilized to image flow and perfusion, changes in blood volume and detect downstream pathological changes but are unable to characterize the changes in the earlier stages of the disease.

Positron Emission Tomogrpahy (PET) has already been accepted as a well established noninvasive diagnostic imaging technique using positron emitting radionuclides for diagnosis, staging/restaging and monitoring of therapy response in oncology. Molecular imaging using PET tracer kinetics provides quantitative *in vivo* assessment of biological and physiological processes involved in the earlier stages of disease progression. Thus PET can be regarded as a promising tool for diagnosis of several diseases where derangements are still at the molecular level and help selecting appropriate modality of treatment. ^18^F reporter probes like fluoropenciclovir (FPCV), fluoroganciclovar (FGCV), etc, have been successfully deployed for imaging herpes simplex virus type 1 - thymidine kinase (HSV1-tk) reporter gene expression.

### Multi-modality molecular imaging

The image fusion through co-registration technique is done using dedicated software. It could enable high resolution morphological CT or MRI images to be registered on functionally informative PET images. However, the problems like motion artifact, complex realignment and computation needed for fusion of independent studies need to be extensively addressed to obtain more informative bimodal or multi-modal images. Integrated instrumentation like clinical CT/PET or, for that matter, development of MRI/PET imaging system may offer a solution to these problems; also, combined use of radionuclides and magnetic probes would permit near-simultaneous MRI and PET imaging. It is also open to choose other combinations out of optical, radionuclide, MRI and CT probes to generate multi-modal images enabling unique information relating to quantification and location of biological events/processes as well as characterization of newly developed imaging probes. The multi-modality images would substantially improve quantification and interpretation of several experimental results.

### Stem cell research

The transplantation of stem cells with damaged tissue had offered tremendous potential for therapeutic applications in a wide range of disease conditions.[19,20] including myocardial infarction and Parkinson's disease. Positron Emission Tomography can be effectively used as sensitive imaging modality for detection as well as tracking of implanted stem cells. The stable transfection of cells with a reporter gene can be serially visualized using a reporter probe and Positron Emission Tomography (PET). This would permit detection, transition and monitoring of the functions of transplanted stem cells.

## Clinical applications in oncology

The Positron Emission Tomography (PET), as already explained, has a unique ability to provide functional as well as metabolic information related to tumor cells. In order to effectively deploy this characteristic, Flourine-18 Fluoro-2-Deoxy Glucose (FDG) has been used to image and characterize cancer cells. In addition, the co-registration of PET and CT or MRI image provides detailed anatomical as well as functional information so as to optimize radiation therapy treatment planning. Integrated PET-CT systems enable not only precise attenuation correction but also provide fusion images. In the Indian scenario, the first PET-CT system was installed at the Tata Memorial Hospital in Mumbai in 2003 and, recently, AIIMS in New Delhi; Army Hospital, New Delhi, has also acquired this facility. The facilities are being predominantly used for oncological applications. Many more centers are in the offing of acquiring PET (CT) systems that are expected to revolutionize the use of PET (CT) in the country as far as its application in radiation oncology is concerned. PET (CT) would be of immense utility in staging, delineation of tumor volume, monitoring of treatment response and detection of recurrence of tumor in wide range of solid tumors. Even though experience related to PET (CT) application in Oncology is very limited from the Indian viewpoint, from whatever limited experience/data is available, it has been shown to improve TNM staging in patients of various cancers. Simultaneously, efficacy of radiation therapy treatment can also be evaluated by estimating Standard Uptake Value (SUV) using PET images. The differentiation between radiation necrosis and malignant metastatic disease in the treatment follow-up can be effectively predicted. In view of this, a concept of Biological Target Value (BTV) has now emerged and is expected to open up a new era of biological conformity. Hypoxic cells within the tumor can also be delineated in a better manner so as to have optimized dose escalation, further improving radiation therapy of cancer cells.

### Pulmonary nodules

The PET-CT has now been successfully deployed as a noninvasive tool for the diagnosis of pulmonary nodules to differentiate between malignant and benign lesions. The PET image generally shows intensive FDG uptake which is more than that in the blood pool activity after about 60 min of injection. The delayed images further improve the results. The benign lesions indicate declining uptake with time, whereas malignant nodules show increase in FDG uptake in time. In case of lung cancers, the PET-CT fusion images may be of immense utility for delineation of nodal disease as well as detection of distant metastasis. PET imaging can be also used as an effective diagnostic tool to rule out presence of metastasis in false positive cases.

### Breast cancer

The PET imaging has assumed significant importance in accurate detection of multifocal disease as well as distant metastasis/lymph nodes in carcinoma of the breast (breast cancer). This is particularly of great clinical importance because in most of the countries, the prevalence of breast cancer in women has shown a rising trend. The PET imaging can also be effectively utilized to assess the response of adjuvant chemotherapy.

### Lymphoma and colorectal cancer

The use of PET imaging in diagnosis, staging and monitoring of therapy response in cases of lymphoma patients has revolutionized the field of oncology. Additional lesions that are not detected by conventional diagnostic techniques like CT can now be delineated and therefore, the efficacy of treatment can be further optimized. In addition, the Whole Body PET imaging can help in modification of therapy in significantly large number of patients. PET images can demonstrate very high predictive value for differentiation between active tumor and fibrosis.

In case of colorectal cancers, FDG PET can have a very high sensitivity for preoperative diagnosis. In addition, PET images can be used to detect recurrence of disease in the follow-up after therapy. The detection of extra hepatic metastasis can be done using PET images, thereby enabling selection of patients who may be fit cases for consideration of partial hepatectomy.

### Head and neck cancer

In order to have optimized treatment planning, lymph node staging in head and neck cases using PET images can be more accurately and comprehensively done. A large number of undetected lesions/lymph nodes could be demonstrated in PET images. In addition, treatment response can be effectively monitored so as to institute appropriate mode of therapy in the follow-up of patients.

### Cancer of gastrointestinal tract

The sensitivity for detection of primary gastric/esophageal cancer as well as metastasis is quite high from specificity viewpoint in FDG PET images. It can particularly help in distinguishing the responsive and nonresponsive disease so as to avoid unnecessary surgery in significant number of patients.

### Osteosarcoma and ovarian cancer

FDG uptake in cases of osteosarcoma can be a good indicator for predicting tumor aggressiveness apart from its utility for enabling guided biopsy so as to select metabolically active tumor site. The ability of PET images to effectively demonstrate therapy response is an added advantage.

In case of ovarian cancer, precise staging of the case is extremely important for effective management, particularly in relatively advanced disease. The sensitivity as well as specificity of PET images for detection of lymph nodes is extremely high as compared to other conventional imaging modalities.

## Conclusion

The emergence of Positron Emission Tomography (PET) has opened up a new era to demonstrate tumor physiology and anatomy. The inherent problems of intra-patient absorption as well as attenuation correction has been taken care of due to technological excellence wherein PET systems are combined with CT to not only channel fusion images but also enable attenuation correction in an approximate manner. This is to emphasize that attenuation correction factors measured by CT are only scaled up for 511 keV and PET-CT enables only segmental attenuation correction. In order to achieve precise attenuation correction, it is advisable to generate attenuation data for transmission scan using 68-Ge source instead of using integrated CT. Dedicated and self-shielded cyclotrons have been developed to provide a large number of radiopharmaceuticals for clinical applications as well as research. Molecular imaging using PET could provide quantitative estimation of biological and physiological processes in very early stages of disease progression. The future of PET imaging lies in depicting the changes at molecular level and hence in selecting appropriate mode of treatment. The application of PET in Oncology has not only altered the concepts used in treatment planning but also has registered its need for monitoring of therapy response. It therefore goes without saying that PET imaging is going to be a diagnostic tool of choice in a large number of clinical applications including Stem Cell research.
